# Supplementary material for: New Insights into the Microbial Profiles of Infected Root Canals in Traumatized Teeth
Source: J Clin Med. 2020 Nov 28;9(12):3877. doi: 10.3390/jcm9123877 (PMC7760719; doi:10.3390/jcm9123877)
Supplement: Supplementary file 1 [file jcm-09-03877-s001.pdf]

Table 1. Detection frequency (%) of species identified by culture and characterisation of isolates by comparing 16SrRNA gene sequences with the database HOMD.

	Species isolated	Trauma [a]	Non-trauma[a]	Total[a]	Trauma[b] (%)	Non-trauma[b] (%)
1	Fusobacterium nucleatum	11	7	18	50,000	24,138
2	Slackia exigua	6	10	16	27,273	34,483
3	Dialister invisus	7	7	14	31,818	24,138
4	Parvimonas micra	3	9	12	13,636	31,034
5	Peptostreptococcaceae infirmum / sulci	2	8	10	9,091	27,586
6	Prevotella nigrescens	4	6	10	18,182	20,690
7	Pseudoramibacter alactolyticus	0	10	10	0,000	34,483
8	Prevotella denticola	4	4	8	18,182	13,793
9	Actinomyces georgiae	2	5	7	9,091	17,241
10	Atopobium rimae	2	5	7	9,091	17,241
11	Peptostreptococcus stomatis	2	5	7	9,091	17,241
12	Actinomyces gerencseriae	3	3	6	13,636	10,345
13	Mogibacterium timidum	2	4	6	9,091	13,793
14	Peptostreptococcaceae yurii	5	1	6	22,727	3,448
15	Selenomonas noxia	5	1	6	22,727	3,448
16	Atopobium parvulum	0	5	5	0,000	17,241
17	Olsenella uli	1	4	5	4,545	13,793
18	Parvimonas sp.	1	4	5	4,545	13,793
19	Porphyromonas gingivalis	0	5	5	0,000	17,241
20	Prevotella sp.	2	3	5	9,091	10,345
21	Alloprevotella tannerae	4	0	4	18,182	0,000
22	Anaeroglobus geminatus	1	3	4	4,545	10,345
23	Campylobacter showae	4	0	4	18,182	0,000
24	Dialister pneumosintes	2	2	4	9,091	6,897
25	Enterococcus faecalis	0	4	4	0,000	13,793
26	Filifactor alocis	0	4	4	0,000	13,793
27	Streptococcus sanguinis	2	2	4	9,091	6,897
28	Actinomyces meyeri	1	2	3	4,545	6,897
29	Campylobacter rectus	3	0	3	13,636	0,000
30	Capnocytophaga sp.	3	0	3	13,636	0,000
31	Olsenella sp.	1	2	3	4,545	6,897
32	Peptoniphilus lacrimalis	1	2	3	4,545	6,897
33	Peptostreptococcaceae nodatum	0	3	3	0,000	10,345
34	Porphyromonas catoniae	3	0	3	13,636	0,000
35	Streptococcus constellatus / intermedius	1	2	3	4,545	6,897
36	Aggregatibacter sp.	0	2	2	0,000	6,897
37	Bulleidia extructa	0	2	2	0,000	6,897
38	Capnocytophaga ochracea	0	2	2	0,000	6,897
39	Cutibacterium acnes	1	1	2	4,545	3,448
40	Eikenella corrodens/Kingella denitrificans	0	2	2	0,000	6,897
41	Leptotrichia hongkongensis	1	1	2	4,545	3,448
42	Mogibacterium	1	1	2	4,545	3,448
43	Olsenella profusa	0	2	2	0,000	6,897
44	Paenibacillus glucanolyticus	0	2	2	0,000	6,897
45	Prevotella intermedia	0	2	2	0,000	6,897
46	Prevotella oralis	0	2	2	0,000	6,897
47	Propionibacterium propionicum	1	1	2	4,545	3,448
48	Pyramidobacter piscolens	0	2	2	0,000	6,897
49	Selenomonas sp.	2	0	2	9,091	0,000
50	Shuttleworthia satelles	1	1	2	4,545	3,448
51	Staphylococcus (troligen epidermidis)	2	0	2	9,091	0,000
52	Actinomyces israelii	0	1	1	0,000	3,448
53	Actinomyces johnsonii	1	0	1	4,545	0,000
54	Actinomyces lingnae	1	0	1	4,545	0,000
55	Actinomyces massiliensis	1	0	1	4,545	0,000
56	Bacillus subtilis	1	0	1	4,545	0,000
57	Campylobacter concisus	0	1	1	0,000	3,448
58	Campylobacter gracilis	1	0	1	4,545	0,000

59	Capnocytophaga sputigena	0	1	1	0,000	3,448
60	Catonella morbi	1	0	1	4,545	0,000
61	Fusobacterium necrophorum	1	0	1	4,545	0,000
62	Gemella bergeri	1	0	1	4,545	0,000
63	Granulicatella adiacens	0	1	1	0,000	3,448
64	Lactobacillus	0	1	1	0,000	3,448
65	Peptoniphilus asaccharolyticus	0	1	1	0,000	3,448
66	Peptostreptococcaceae [XI][G-5] bacterium	1	0	1	4,545	0,000
67	Peptostreptococcaceae [XI][G-7] yurii subspp. yurii & margaretiae	1	0	1	4,545	0,000
68	Prevotella baroniae	0	1	1	0,000	3,448
69	Prevotella buccae	0	1	1	0,000	3,448
70	Prevotella melaniogenica	0	1	1	0,000	3,448
71	Prevotella oulorum	1	0	1	4,545	0,000
72	Propionibacterium acidifaciens	0	1	1	0,000	3,448
73	Propionibacterium avidum	0	1	1	0,000	3,448
74	Staphylococcus epidermidis / caprae	0	1	1	0,000	3,448
75	Staphylococcus pasteuri	1	0	1	4,545	0,000
76	Staphylococcus warneri	1	0	1	4,545	0,000
77	Streptococcus intermedius	0	1	1	0,000	3,448
78	Streptococcus anginosus	0	1	1	0,000	3,448
79	Veillonella dispar / parvulva	0	1	1	0,000	3,448

Table 2. Bacterial association to trauma and non-trauma teeth presented at species level.

Phylum	Genus	Species	log2Fold Change	*p*-adj
Firmicutes	Mogibacterium	timidum	-7.733251	0.0000000
Firmicutes	Stomatobaculum	sp._HMT_910	-9.750942	0.0000000
Firmicutes	Selenomonas	noxia	9.230914	0.0000015
Firmicutes	Lactobacillus	casei	-8.647475	0.0000000
Firmicutes	Veillonellaceae_[G-1]	bacterium_HMT_132	6.311425	0.0001167
Firmicutes	Selenomonas	sputigena	-5.713459	0.0000573
Firmicutes	Peptostreptococcaceae_[XI] [G-7]	yurii_subspp._yurii_&_ margaretiae	13.193901	0.0000000
Firmicutes	Peptoniphilus	lacrimalis	5.684783	0.0006669
Firmicutes	Peptostreptococcaceae_[XI] [G-9]	brachy	6.487365	0.0001905
Firmicutes	Streptococcus	vestibularis	-6.739119	0.0000000
Firmicutes	Mycoplasma	salivarium	-6.612241	0.0000005
Firmicutes	Johnsonella	ignava	7.926672	0.0000125
Firmicutes	Paenibacillus	glucanolyticus	-13.882510	0.0000000
Firmicutes	Veillonella	dispar	-4.289847	0.0052933
Firmicutes	Peptostreptococcaceae_[XI] [G-4]	bacterium_HMT_369	7.587072	0.0000274
Spirochaetes	Treponema	maltophilum	8.758014	0.0000002
Bacteroidetes	Porphyromonas	endodontalis	-8.874290	0.0000000
Bacteroidetes	Bacteroidetes_[G-3]	bacterium_HMT_365	-9.573662	0.0000000
Bacteroidetes	Prevotella	sp._HMT_317	-6.935651	0.0000443
Bacteroidetes	Porphyromonas	sp._HMT_275	6.074244	0.0003361
Bacteroidetes	Bacteroidales_[G-2]	bacterium_HMT_24	5.888751	0.0001110
Bacteroidetes	Bacteroidetes_[G-5]	bacterium_HMT_51	8.562230	0.0000055
Bacteroidetes	Prevotella	nanceiensis	-6.682456	0.0000000
Bacteroidetes	Porphyromonas	gingivalis	-6.900814	0.0000000
Bacteroidetes	Prevotella	sp._HMT_313	-7.537308	0.0000000
Bacteroidetes	Prevotella	sp._HMT_292	-4.873577	0.0005656
Bacteroidetes	Prevotella	nigrescens	4.470768	0.0048599
Bacteroidetes	Prevotella	intermedia	-11.970001	0.0000000
Bacteroidetes	Alloprevotella	sp._HMT_473	-7.560106	0.0000000
Bacteroidetes	Prevotella	melaninogenica	-10.595443	0.0000000
Actinobacteria	Rothia	dentocariosa	-4.342347	0.0037499
Actinobacteria	Actinomyces	oris	6.913926	0.0000188
Actinobacteria	Corynebacterium	matruchotii	4.342142	0.0056922
Actinobacteria	Rothia	mucilaginosa	-6.811720	0.0000001
Actinobacteria	Atopobium	sp._HMT_199	-10.051236	0.0000000
Actinobacteria	Olsenella	uli	-5.396915	0.0001584
Proteobacteria	Escherichia	coli	-8.420180	0.0000000
Proteobacteria	Haemophilus	parainfluenzae	-4.978336	0.0001306
Proteobacteria	Yersinia	pestis	-9.918709	0.0000000
Proteobacteria	Pseudomonas	fluorescens	-5.958878	0.0000017
Proteobacteria	Campylobacter	showae	6.682366	0.0000230
Proteobacteria	Campylobacter	concisus	-10.118541	0.0000000
Proteobacteria	Cardiobacterium	hominis	5.582932	0.0010912
Fusobacteria	Fusobacterium	nucleatum_subsp._ polymorphum	5.423556	0.0026090
Fusobacteria	Fusobacterium	hwasookii	-6.694746	0.0000045
Fusobacteria	Fusobacterium	sp._HMT_203	6.654830	0.0000787
Synergistetes	Pyramidobacter	piscolens	-8.983015	0.0000000

Synergistetes	Fretibacterium	fastidiosum	-4.666563	0.0053617

Different HMT sorted based on the Phylum to find a specific organism easily. The color differences to easily separate the high abundant from the low abundant in Trauma samples. If the log2FoldChange is above ZERO (blue), meaning that the HMTs were more abundant in Trauma samples and below ZERO (orange) HMTs were more abundant in Non-Trauma samples.
